# *Empedobacter brevis* Bacteremia in a Patient Infected with HIV: Case Report and Review of Literature

**DOI:** 10.1155/2015/813528

**Published:** 2015-10-15

**Authors:** Syed Bokhari, Naeem Abbas, Manisha Singh, Richard B. Cindrich, Cosmina Zeana

**Affiliations:** Division of Infectious Diseases, Bronx-Lebanon Hospital Center, Bronx, NY 10457, USA

## Abstract

Clinical disease caused by *Empedobacter brevis* (*E. brevis*) is very rare. We report the first case of *E. brevis* bacteremia in a patient with HIV and review the current literature. A 69-year-old man with human immunodeficiency virus (HIV) and CD4 count of 319 presented with chief complaints of black tarry stools, nausea and vomiting for 2 days. Physical exam was significant for abdominal pain on palpation with no rebound or guarding. His total leukocyte count was 32,000 cells/*μ*L with 82% neutrophils and 9% bands. Emergent colonoscopy and endoscopic esophagogastroduodenoscopy showed esophageal candidiasis, a nonbleeding gastric ulcer, and diverticulosis. Blood cultures drawn on days 1, 2, and 3 of hospitalization grew *E. brevis*. Patient improved with intravenous antibiotics. This case is unusual, raising the possibility of gastrointestinal colonization as a source of the patient's bacteremia. In conclusion, *E. brevis* is an emerging pathogen that can cause serious health care associated infections.

## 1. Introduction

*Empedobacter brevis* (*E. brevis*) is a Gram-negative bacillus that belongs to the Flavobacteriaceae family. It is commonly found in soil, water, raw meat products, and hospital environments [1, 2]. However, clinically significant disease due to* E. brevis* is very rare. We report the first case of* E. brevis* bacteremia in a patient with HIV and review the current literature.

## 2. Case Summary

A 69-year-old Panamanian man with history of human immunodeficiency virus (HIV) infection with CD4 count of 319 presented from a long term facility with chief complaint of black tarry stools for 2 days associated with malaise, nausea, and vomiting for one week. One week prior to the presentation, he had received corticosteroid injections in both knees for osteoarthritis. Three months prior to admission he had undergone left eye cataract surgery without complications. There was no history of recent travel or active substance abuse. In the emergency department, vital signs included temperature of 97.6 degrees Fahrenheit, respiratory rate of 14, pulse of 92, blood pressure of 117/87 mmHg, and oxygen saturation of 99% on room air. The oropharynx was clear, cardiac and respiratory examination was normal, abdominal examination was significant for mild pain at palpation diffusely, and there were normal bowel sounds and no rebound or guarding. Both knee joints were normal on examination with no signs of inflammation, tenderness, or restriction of movement. No skin lesions or rash was noted. The remainder of the examination was unremarkable. A complete blood count revealed anemia and elevated total leukocyte count. The hemoglobin level on admission was 10.7 g/dL and hematocrit was 34% which dropped to 7.4 g/dL and 23.4%, respectively, after fluid resuscitation. The total leukocyte count was 32,000 cells/*μ*L with the differential showing 82% neutrophils, 9% bands, and 2% lymphocytes. The coagulation profile was significant for an elevated International Normalized Ratio (INR) of 6.7 and a normal activated partial thromboplastin time of 33.5 seconds. Renal function was abnormal with elevated creatinine of 2.2 mg/dL. Liver function tests and the remainder of electrolyte levels were normal. Due to concern for active gastrointestinal tract bleeding, the patient was admitted to the intensive care unit and started on intravenous fluids. He also received fresh frozen plasma (FFP) to correct the coagulopathy and 1 unit of packed red blood cells. An emergent colonoscopy and endoscopic esophagogastroduodenoscopy showed esophageal candidiasis, a nonbleeding gastric ulcer, and diverticulosis. The blood cultures drawn on days 1, 2, and 3 of hospitalization showed growth of Gram-negative bacilli and antibiotic therapy with piperacillin-tazobactam 3.375 mg every 6 hours was initiated while awaiting final identification of the organism. The Gram-negative bacilli were identified as* E. brevis*, sensitive to fluoroquinolones, trimethoprim-sulfamethoxazole, tigecycline, polymyxin B, and piperacillin-tazobactam. Investigations aiming at identification of the source of bacteremia included a transthoracic echocardiogram which showed no vegetation. In the absence of any signs or symptoms of inflammation of the knee joint, the primary team, the consulting rheumatologist, and orthopedic surgeon decided against performing an arthrocentesis. In view of recent cataract surgery, an ophthalmologic examination was performed, which was unremarkable. Piperacillin-tazobactam was continued and repeat blood cultures on day 4 showed no growth. The patient responded to therapy and his total leukocyte count trended down towards normal level. The patient received piperacillin-tazobactam for 11 days and was discharged back to a skilled care facility on oral ciprofloxacin to complete two weeks of antibiotics. He was admitted subsequently to our facility for reasons unrelated to this admission and repeat blood cultures showed no growth.

## 3. Discussion

*E. brevis*, formerly known as* Flavobacterium brevis*, are short, nonmotile, Gram-negative rods that grow easily on routine culture media. They are obligate aerobes which form a yellow colony when grown on solid media. Most strains grow at 37°C and all strains grow at 30°C.* E. brevis* are positive for catalase, oxidase, and phosphatase and produce indole [1, 2]. These organisms are widely distributed in soil, water, and plants but remain an unusual cause of infections in humans.* E. brevis* can also be found in the hospital environment, leading to rare cases of nosocomial infections [3–5]. Usually,* E. brevis* is susceptible to several classes of antibiotics including beta-lactams, fluoroquinolones, and aminoglycosides. However, treatment of infections caused by* E. brevis* can be complicated by the presence of a chromosome-encoded Ambler class B beta-lactamase, which confers decreased susceptibility to extended spectrum cephalosporins and carbapenems [6].

A review of literature revealed only four reports of* E. brevis* infections.

A series of eleven patients with* E. brevis* endophthalmitis after uncomplicated cataract surgery was reported from Germany [4]. The patients had undergone cataract extraction surgery, performed by the same surgeon, 1–6 days before presentation. All 11 subjects were found to have* E. brevis* growing from intraocular cultures. They were treated with intravitreal vancomycin and amikacin in addition to ophthalmologic interventions and had good clinical outcomes. Culture of the solutions used for irrigation, intraocular lenses, tap water, and surgical instruments did not grow the organism. A problem with the sterilization process was considered the possible cause of this outbreak.

*E. brevis* was implicated as a cause of cellulitis in an 83-year-old patient from Japan [7]. The patient presented with erythema, blisters, and purpura of her right foot. Skin biopsy showed leukocytoclastic vasculitis and culture grew* E. brevis*. The infection resolved after treatment with intravenous minocycline.

*E. brevis* has also been reported to cause ventriculostomy-associated infections [3]. A retrospective review of 28 cases of meningitis in patients with ventriculostomy from Taiwan showed one infection to be caused by* E. brevis*.

Most recently, a case of knee cellulitis with bacteremia was reported in a patient who had undergone right knee replacement 6 weeks prior to presentation [5]. The infection was successfully treated with intravenous levofloxacin for 10 days.

## 4. Conclusion

*E. brevis* is an emerging pathogen with potential to cause infection in an immunodeficient host. We report the first case of* E. brevis* bacteremia in a patient with HIV. There has only been one other reported case of* E. brevis* bacteremia in a patient who presented with cellulitis [5]. Our case is unusual, raising the possibility of gastrointestinal colonization as a source of the patient's bacteremia. In conclusion,* E. brevis* is an emerging pathogen that can cause serious health care associated infections. The relationship between* E. brevis* and immunodeficiency remains to be further evaluated.
